# Perceived Impacts of Partners’ Other Relationships on Oneself in Consensual Nonmonogamy

**DOI:** 10.1007/s10508-024-02823-7

**Published:** 2024-03-04

**Authors:** Jennifer Arter, Sacha S. Bunge

**Affiliations:** Psychology Department, San Francisco State University, San Francisco, CA 94132 USA

**Keywords:** Consensual nonmonogamy, Polyamory, Metamours, Romantic relationships, Sexual orientation

## Abstract

Existing research suggests a number of both costs and benefits to oneself that can occur as a result of partners’ other relationships in consensual nonmonogamy (CNM), but such costs and benefits have not previously been systematically cataloged. Using reflexive thematic analysis, we present themes derived from semi-structured interviews with 51 individuals (63% white, 55% nonbinary/genderqueer/non-cisgendered, and 77% LGBQ) who have practiced CNM, describing the costs and benefits to themselves that they perceive as a result of their partners’ other relationships. Themes describing costs include experiencing difficult feelings (e.g., jealousy), having less with a partner (e.g., less of a partner’s time) than one would like, difficulties or conflict within one’s own relationship, and difficulties or conflict as a result of interactions between metamours (individuals who share a partner). Themes describing benefits include experiencing positive feelings (e.g., compersion), benefiting from a partner getting needs met in other relationships, strengthening of or pleasurable interactions within one’s own relationship, enjoyable or beneficial relationships or interactions between metamours, and personal growth as a result of sharing partners with others. We note that these costs and benefits roughly mirror each other, suggesting that they may reflect the positive and negative sides of some fundamental aspects of CNM. Each of our themes also contains a rich range of elements that suggest avenues for future research. Our results suggest that CNM relationships are complex and multi-faceted, and that further research could fruitfully examine the circumstances that influence whether individuals experience their partners’ other relationships positively or negatively.

## Introduction

Consensual nonmonogamy (CNM; Conley et al., 2013) is a term that encompasses a range of relationship practices (e.g., polyamory, swinging, or open relationships; Barker & Langdridge, 2010; Cohen, 2015; Ferrer, 2018a; Frank & DeLamater, 2010). Common across different forms of CNM is that it often involves sharing partners with others, such that individuals do not have exclusive access to their partners’ romantic and/or sexual time, energy, and attention. The literature to date suggests that this sharing has an impact on those who practice CNM, influencing their relationships and their feelings about their relationships both positively and negatively.

Work to date that has begun to describe the perceived costs and benefits of CNM relationships has often focused on either the perceived benefits of participants’ own multiple relationships (Cohen, 2015; Moors et al., 2017; O’Byrne & Haines, 2021; St. Vil et al., 2021; Wood et al., 2021a, 2021b) or on specific, predetermined variables (e.g., relationship satisfaction or jealousy; see also Balzarini & Muise, 2020; Rubel & Bogaert, 2015). Thus far, there is no research that has attempted to comprehensively catalog the costs and benefits that individuals might perceive for themselves as a result of their partner(s) having multiple consensual relationships. It might be tempting to predict that all of the perceived benefits of CNM come from one’s own multiple relationships, and that one’s partner(s) also having multiple relationships is perceived as a cost that one tolerates in exchange. But the literature suggests that the reality is not so simple, and that there are likely both benefits and costs that result from partners’ other relationships (for a similar argument, see Watson & Lubrano, 2021).

This suggestion comes first from research examining whether relationship satisfaction in pairs of CNM partners is related to their extra-dyadic activities, often specifically the ways in which other partners are meeting their sexual and/or emotional needs. This research suggests that these influences may be complex (for reviews, see Balzarini & Muise, 2020; Moors et al., 2017). For example, Muise et al. (2019) reported that need fulfillment in one relationship could enhance one’s own satisfaction with another relationship, whereas Michell et al. (2014) found that these variables were significantly but weakly related in the opposite direction. Hosking (2013) found that gay men in open relationships who perceived that they and their primary partners benefited equally from their respective extra-dyadic activities reported greater relationship satisfaction, whereas a perception of unequal benefit (in either direction) was associated with lower relationship satisfaction. Such findings hint that there are likely a complicated set of positive and negative ways in which CNM relationships can influence each other.

### Benefits of Partners’ Other Relationships

Studies have documented participants’ qualitative reports that one relationship can support, enhance, or strengthen another (Bartell, 1970; de Visser & McDonald, 2007; O’Byrne & Haines, 2021; Ramey, 1975; Thouin-Savard, 2021; Watson, 1981; Wood et al., 2021a; and for reviews, see Jenks, 1998; Rubel & Bogaert, 2015); that having an open relationship could make partners feel closer or feel more love for each other (Cohen, 2015; Thouin-Savard, 2021; Wolfe, 2003); that having multiple partners can add excitement or inspire intimate interactions in a given relationship (Bartell, 1970; Deri, 2015; St. Vil et al., 2021); that greater “stability” (Ramey, 1975) or increased material and labor resources (Sheff, 2014) can come via partners’ other relationships; that having other partners can be a way to manage differences in sexual desires (Deri, 2015; McLean, 2004; O’Byrne & Haines, 2021; St. Vil et al., 2021; Wood et al., 2021a); or that CNM can contribute to both personal growth and growth in relationships (Wood et al., 2021a). Given that personal growth appears to be one of the major perceived benefits of engaging in CNM (see Moors et al., 2017), such growth might also result in benefits to partners (Thouin-Savard, 2021).

Another line of research suggests that one specific positive influence on oneself of partners’ other relationships is the experience of compersion: happiness or pleasure in response to a partner’s enjoyment of other relationship activities (Deri, 2015; Duma, 2009; Flicker et al., 2021; Mogliski et al., 2019; Ritchie & Barker, 2006; Rubinsky, 2018; Thouin-Savard, 2021; Wolfe, 2003). Compersion may be relatively common (Wolfe, 2003) and can involve personal enjoyment and sexual arousal (Deri, 2015; Flicker et al., 2021; Ramey, 1975; Thouin-Savard, 2021) as well as happiness stemming from empathically sharing in partners’ positive feelings about their other partners (Flicker et al., 2021; Thouin-Savard, 2021). Moreover, experiencing compersion might result in broader benefits to oneself. Thouin-Savard (2021) reports that participants experienced compersion as enhancing their self-growth, and in one quantitative study, compersion was positively correlated with relationship satisfaction among women in open relationships (Aumer et al., 2014).

And finally, a handful of studies have documented the potential for benefits to oneself as a result of interactions with partners’ other partners (i.e., one’s own metamours). Weitzman (2006) suggests that metamours can develop trust and goodwill that can help to smooth conflict, and Bove (2017) describes reports of cooperation and collaboration among metamours. Metamour connections can also become personal ones; Ritchie and Barker (2006) report participants describing their metamour connections fondly, and Bove (2017) and Thouin-Savard (2021) document metamour friendships and family-like relationships. Sheff (2014) documents ways in which polyamorous families collaborate in household tasks and child-rearing, often including bonds of affection and mutual assistance between metamours, and Watson and Lubrano (2021) document historical accounts of collaboration and assistance among metamours.

### Costs of Partners’ Other Relationships

There are also findings suggesting some of the perceived costs or difficulties to oneself that can arise from a partner’s other relationships. Some studies describe small numbers of participants who reported that being in an open relationship made them feel “further away” from a partner (Cohen, 2015); that CNM had resulted in more fighting, greater instability, or a diminished bond with a partner (Wolfe, 2003); that practicing CNM had threatened a relationship or led to separation (Rubel & Bogaert, 2015); or that conflict could arise particularly at the start of nonmonogamous relationships (Watson & Lubrano, 2021). Wood et al. (2021b) found that when an individual reported higher sexual need fulfillment with a second partner, that individual’s first partner reported lower sexual and relationship satisfaction on average, suggesting that a partner’s sexual activities with others could detract from one’s own relationship with that partner.

Another perceived cost of CNM relationships is the potential to experience difficult emotions, often described using the term jealousy (e.g., Bartell, 1970; Cohen, 2015; de Visser & McDonald, 2007; Deri, 2015; Ritchie & Barker, 2006; Rubel & Bogaert, 2015) but perhaps better understood as a range of related unpleasant feelings (e.g., McLean, 2004; Rubinsky, 2018; Wolfe, 2003). While the extent to which painful emotions are a problem for those who practice CNM appears to vary (Bergstrand & Williams, 2000; Ramey, 1975), difficult feelings clearly do arise in CNM relationships, are experienced by some as unhealthy or problematic (Rubinsky, 2018), and can negatively impact relationship satisfaction (Rubinsky, 2019). Further, such feelings may be a cost not only because they are unpleasant and might impact happiness in one’s relationship, but also because they require time and energy to manage (de Visser & McDonald, 2007; McLean, 2004). Watson (1981) and Rubinsky (2018) describe participants’ reports of the time and energy they spend reflecting on and learning to navigate their own feelings of jealousy and assisting partners who are feeling jealousy, while Watson (1981) reports participants’ descriptions of the adjustments they sometimes make in their activities with a secondary partner in order to allay the jealousy of a primary partner.

A related cost of CNM relationships is the more general time and energy spent investing in multiple relationships and navigating complex interpersonal situations (e.g., Deri, 2015; Sheff, 2014; Weitzman, 2006). Rubinsky (2018) documents participants’ reports of the feeling of “missing out” on desired activities with a partner, and Wolfe (2003) reports that over half of participants expressed a desire to spend more time with a partner. Cohen (2015) reports that many participants named time constraints or time management as difficulties in CNM relationships, and Ramey (1975) found that many individuals in “sexually open” friend groups endorsed lack of time, scheduling difficulty, and sleeping arrangements as problems. And time that is spent with partners can end up being spent negotiating complex situations and feelings; one sample of participants in open marriages reported spending an average of an hour per day discussing their open relationship (Watson, 1981). Cohen (2015) found that many participants named “communication issues” as difficulties, and Ramey’s (1975) participants frequently endorsed difficulties described as “situational complexities” and “daily tensions.” These findings suggest that time costs, both missing out on time with a partner and spending one’s time with a partner discussing and negotiating, may be an important cost of partners’ other relationships.

Finally, there is some suggestion in the literature of the costs that might result from interactions between metamours. Rubinsky (2018) and Deri (2015) document that individuals can feel that they are in competition with a metamour, in particular when they perceive a metamour as being either similar to themselves or more physically attractive than themselves (Deri, 2015) or when a partner exhibits excitement about a new metamour (Rubinsky, 2018). Rubinsky (2018) and Thouin-Savard (2021) also document the problem of simply not liking a particular metamour, and Rubinsky (2018), Weitzman (2006), and Thouin-Savard (2021) describe the potential for a metamour to attempt to interfere with or end one’s own relationship with a shared partner.

As this review shows, evidence suggests a range of positive and negative perceived effects on oneself of partners’ other relationship activities, which may operate in complex ways. However, so far, this evidence exists in piecemeal fashion; no study has yet attempted to catalog perceptions of the ways in which partners’ other relationship(s) affect oneself. In this paper, we set out to comprehensively catalog the range of perceived impacts on oneself, both positive and negative, of one’s partner(s) having other sexual and/or romantic relationships, as described by individuals with a lived experience of consensual non-exclusivity.

## Method

### Participants

Participants were 51 adults who reported an average age of 37.1 years (SD = 9.6); three participants did not report their age. Participants reported from 3 to 50 years of experience in CNM relationships; the majority reported 10 or more years of experience. Asked to report their ethnicity, 32 reported white/European, seven Black/African American, four Asian, four Latinx, and four mixed or other ancestries. Twenty-eight reported their gender as nonbinary, genderqueer, or other non-cisgendered, 18 reported woman/female, and four reported man/male (one did not report their gender). Thirty-five reported bisexual, pansexual, omnisexual, or queer orientations; 10 reported heterosexual or mostly heterosexual; four reported gay/lesbian/homosexual; and two reported “other” or declined to respond (for a more detailed presentation of these demographic characteristics, see Arter & Bunge, 2023).

All participants had engaged in multiple concurrent sexual and romantic/emotional relationships. Although participants were not asked to name the type of CNM they practiced, many were asked what terms they preferred the interviewer to use: 14 had no preference and two declined to identify with any label; nine preferred “poly” or “polyamorous” and 11 indicated that this term worked for them; six preferred “nonmonogamous” and one indicated that this term worked for them; one preferred “relationship anarchist”; and seven were not asked about preferred terms.

### Procedure

Note that a previous qualitative study has been published based on a different subset of data from this sample, and our methods are similarly described in that paper (Arter & Bunge, 2023). The aim of data collection was to explore in an open-ended fashion CNM individuals’ thoughts and feelings about their metamours (i.e., their sexual/romantic partners’ other sexual/romantic partners), their interactions with their metamours, and their partners’ relationships with metamours. Participants were recruited from February 2019 through June 2020 via snowball method and constitute a convenience sample. The first author began recruitment through personal contacts in local CNM communities (in person or via email), and participants were asked to share the study invitation with others. Participants were not recruited through any specific organizations or online fora. We aimed to recruit individuals who had experience interacting with metamours, and therefore, we sought individuals with 5 or more years of experience in CNM relationships, who had gotten to know at least one metamour or who had had at least two partners who had gotten to know each other. Participants with fewer than 5 years of experience with CNM were included in our sample in four cases, due to their extensive experience with metamours (i.e., having spent relatively large amounts of time with and having had impactful interpersonal experiences with metamours; these participants each had 3–4 years’ experience in CNM).

Participants gave informed consent, including consent to specific uses of the audio-recorded interviews (e.g., publication of direct quotes), before data collection began. Interviews were between 48 and 109 min in length and were all conducted by the first author either in person, by phone, or via Zoom. Participants completed a set of demographic questions and then a set of opening questions. Following this, they were given a list of topics related to metamour relationships, which had been created by the first author based on personal experiences and informal conversations with others, and which included the following topic areas: feelings about metamours, communication with metamours, meeting and spending time with metamours, privacy and information-sharing, calendaring and time management, difficult situations, and dating metamours. Our areas of interest were broad, and we anticipated that different individuals would have different kinds of experiences with metamours; therefore, participants chose which of our topic areas they wanted to talk about to guide the interview and were asked a set of questions about each of the topics they chose. The interviews were semi-structured, with participants being explicitly encouraged to follow their own train of thought (see, e.g., Giorgi, 2012; Polkinghorne, 1989).

### Analysis

Transcription of interviews and reflexive thematic analysis were completed by the first author (Bazeley & Jackson, 2013; Braun & Clarke, 2021; Saldaña, 2013) using Transana Basic, version 3.21. Among the developed strategies for conducting qualitative analysis of rich data, reflexive thematic analysis was most appropriate for our purposes because it provided a balance of structure and flexibility appropriate to a broad, inductive exploration of themes constructed from a relatively large body of wide-ranging and complex interview data (Braun & Clarke, 2006, 2021). In reflexive thematic analysis, the researcher first expansively explores broad data via grouping of like quotes, concurrent with and followed by development of themes that describe “repeated patterns of meaning” (Braun & Clarke, 2006, p. 86) across sets of quotes; this process is recognized as inherently subjective, active, and exploratory and does not require multiple coders but does require transparency regarding the researcher’s work process, which we provide next (Braun & Clarke, 2006, 2021; Saldaña, 2013). For a detailed description of the preliminary stages of data processing, see Arter and Bunge (2023). Briefly, we began with the aim of expansively exploring both explicitly described experiences with and feelings about metamours and implicit patterns among responses noted by the researcher, via a broad first-pass analysis of our entire data corpus (Bazeley & Jackson, 2013; Braun & Clarke, 2006, 2021; Saldaña, 2013). The topic of the current paper was one of several generated in the course of this first pass. After all of the interviews had been transcribed and the first-pass analysis was completed, the set of data that were relevant to the current paper’s topic was examined again in detail, with the following aims: to exhaustively explore all relevant participant statements and combine/split them into like sets; to examine these emerging sets of quotes in order to describe their essential similarities and differences and thereby iteratively organize them into themes; to search for and note associations and overlap between distinct themes and their specific elements; and to develop top-level conceptual understandings and descriptions via reflection and interpretation (Bazeley, 2009; Bazeley & Jackson, 2013; Braun & Clarke, 2006; Corbin & Strauss, 1990; Saldaña, 2013). The first author generated the themes; the second author reviewed all themes with their associated sets of quotes, and any disagreements were resolved via discussion (Bazeley, 2009; Bazeley & Jackson, 2013; Corbin & Strauss, 1990; Saldaña, 2013). We report the number of participants whose statements contribute to each theme, as a way of placing themes in context with regard to how widely they are represented in this dataset (Bazeley, 2009; Bazeley & Jackson, 2013; see, e.g., Sizemore & Olmstead, 2018; St. Vil et al., 2021).

## Results

Results are divided into two sections. In the first section, we present four themes describing perceived costs to oneself of partners’ other relationships. In the second section, we present five themes describing perceived benefits to oneself of partners’ other relationships. All participants are represented in at least one of the “benefits” themes, and all but one participant are represented in at least one of the “costs” themes. See Table 1 for a summary of all of the themes and their major elements. Participant statements informing these themes were drawn from throughout the interviews, and in particular, many relevant statements were obtained in response to some of the opening questions as well as in response to questions on the topics of feelings about metamours and privacy and information-sharing (see Appendix 1).

**Table 1:** Summary of themes describing perceived negative and positive effects on oneself of partners' other relationships

Themes describing difficulties/costs
Theme 1: Struggling with unpleasant feelings
Comparisons between self and metamour
Comparisons between own and other relationship
Insecurity, inadequacy, envy, jealousy, fear
Unpleasant feelings as just part of relationships
Unpleasant feelings as habits of monogamous culture
Theme 2: Having less with a partner because of their other relationships
Having less of a partner's time than one would like
Feeling left out of or missing out on specific activities
Wanting activities/commitments that are not available
Theme 3: Difficulties in or undesired changes to own relationship
Partner preoccupied/upset by other relationship
Partner wants help with difficulties in other relationship
Partner changes/ends own relationship for another one
Partner's other relationship leads self to change/end relationship
Metamour's actions have a negative impact on own relationship
Theme 4: Difficulties related to interactions between metamours
Time and energy spent navigating connections with metamours
Disliking, not getting along with a metamour
Sadness or grief over loss of a metamour relationship
Specific difficulties arising in metamour interactions
Difficulties between metamours leading to difficulties with partner

Themes describing positives/benefits
Theme 5: Enjoyment of compersion
Feeling "happy for" partner's other relationship activities
Vicarious enjoyment of partner's other relationship activities
Theme 6: Benefits from partners getting needs met by others
Needs that oneself cannot or does not want to meet
More free time to do other things or be alone
Collaborating with metamour to meet partner's needs
Theme 7: Benefits to own relationship from partners' other relationships
Partner's improved happiness/skills benefit self
Examining own wants by observing partner's other relationship
Enjoyable interactions stemming from partner's other relationship
Theme 8: Benefits as a result of interacting with metamours
Metamours as friends, community, or partners
Partners' preferences result in enjoyable metamour relationships
Enjoyment from interacting with metamour
Metamours directly benefiting each others' relationships
Metamours as general resources for each other
Theme 9: Opportunities for personal growth
Emotional growth, cultivating self-love/security
Becoming more self-aware, better at articulating needs

### Section 1: Perceived Difficulties or Costs to Oneself Stemming from Partners’ Other Partners/Relationships

#### Theme 1: Struggling with Unpleasant Feelings, Often Stemming from Comparison (n = 38)

Participants described struggling with unpleasant feelings related to sharing a partner. We note that this theme in some ways intersects with each of the other themes that describe costs to oneself (Themes 2, 3, and 4); any difficult situation in a relationship will likely involve unpleasant feelings. What is included in this theme are negative feelings that are not clearly associated with any of the specific difficulties or outcomes described in other themes, and whose main costs are their painfulness and the time and energy spent handling them.

Difficult feelings were often described as stemming from comparing oneself to a metamour or from a fear of being compared unfavorably to metamours by partners.

(Interviewer: When your existing partner starts to date a new person, just first of all, like what is that like for you?) It’s always a little hard. It’s always a little bit hard, I always feel nervous that, less so now, but I always feel a little nervous that they're going to be hotter than me, they're going to be more interesting than me, they're going to be more datable than me... (51)

Similarly, participants described difficult feelings stemming from noticing differences between one’s own relationship and that between one’s partner and metamour.

I mean I don’t always tell [metamour] that like, I’m feeling jealous or resentful. That’s hard to say. I do feel those things, especially when... my partner’s like, more affectionate in ways that maybe we’re struggling with, or if I know they’re having sex and we’re not. (49)

However, sometimes negative feelings were mentioned in a more general way, described with terms such as insecurity, inadequacy, envy, or jealousy, and were occasionally described as a “fear of the unknown” with regard to new metamours or metamours oneself has not met.

Well, yes I have definitely experienced jealousy. I’ve also found out about myself to check if it’s envy or it’s jealousy, or if it’s longing. Because those things feel very similar. (44)

And if you don’t do that [meet metamours]... then these people are essentially strangers, and then it’s really, and then it creates a lot of fear, fear of the stranger... (1)

Negative feelings were sometimes described as a basic reality of human relationships, and sometimes were described as arising partly from habits of thought learned from monogamous culture.

I think jealousy, for me, is the strong fear that you are fundamentally replaceable. And therefore anybody who is a better candidate than you is a threat... that’s just how it feels for me, but coincidentally that is also how we construct the narrative of monogamy... this idea of like, you are looking for the best person that you can find, right?.... It’s a very competitive view of people.... In that paradigm, it’s very scary to meet people who are better than you at other things, because it’s easy to compare yourself... (29)

#### Theme 2: Having Less with a Partner, Because of the Existence of the Partner’s Other Relationships (n = 26)

Participants described sometimes having less of a partner’s time than they would like because of the partner’s other relationships or dating activities, being left out of or missing out on particular events or activities because of a partner spending time with a metamour, or wanting particular commitments or relationship activities that are not available to oneself because of a partner’s other relationship commitments or activities.

...time is like the biggest resource, I think. For me at least, when I’m in a serious relationship with somebody, like I really want to spend a lot of time with them. And if they’re in a serious relationship with somebody else too, then they want to spend a lot of time with both people, and so there can be some tension there. (21)

I think the hardest thing I’ve experienced with a metamour is like, if I know them, and then my partner and they do something cool that I would like to do, I’m like, why didn’t you invite me? When you know that I enjoy these things too? Yeah, so that’s been I think the hardest thing, is feeling left out. (41)

#### Theme 3: Difficulties in or Undesired Changes to One’s Own Relationship (n = 39)

Participants talked about possible relationship difficulties or unwanted changes to one’s own relationship that can stem from a partner’s other relationship(s); these were things that participants described as having happened to themselves, things that they described having feared would happen, or things that they had seen happen to others.

Participants described ways in which difficulties between a partner and metamour could affect that partner in such a way as to indirectly lead to difficulties in one’s own relationship, when that partner is consistently upset about, preoccupied by, or spending a lot of time dealing with difficulties in another relationship.

I was jealous a bit [of partner], when she was with [metamour], and it was because, like that relationship was not healthy for her. And she was like, sort of withdrawing from me, and they were having a lot of trouble. So I think that’s one situation. Yeah if something’s like, bringing my partners down but they’re like putting a lot of energy in that direction anyways, then that can be hard. (23)

Participants also described time and energy spent navigating situations in which a partner wants to talk about or get help with difficulties in another relationship. This was often described as a mild issue, but was occasionally described as involving a person mediating conflicts between their partner and metamour, which could become a larger problem, and intersects with Theme 4, “difficulties related to interactions between metamours.”

...there are some dates I have with some of my partners where it’s like, all we talk about is a fight that they had recently with their person, and that’s not a bad thing, and it happens... [but] after like the 5th week of that happening on our once-a-week or twice-a-week date night, it was just like, it just got a lot. It got really draining. (35)

Participants described having experienced or fearing situations in which developments or decisions within another relationship could lead a partner to reevaluate, change, or end one’s own relationship, or when a partner’s choice of or activities with other partners could lead oneself to reevaluate, change, or end one’s own relationship with that partner.

...[partner] and I had been dating not monogamously for like [amount of time], when he met someone, and then they decided to become monogamous, right?.... (Interviewer: Wow, how is that?) Hard, it sucked... (24)

...[partner] started dating somebody that I had dated. And that person, I felt, did not treat me well.... And I came to realize that that was a deal-breaker.... and so I removed myself from the equation. (Interviewer: Right, and left the relationship.) Yeah. (04)

And finally, some participants described fearing or having experienced ways in which a metamour could do things that have a negative impact on one’s own relationship, for example, by criticizing oneself to a shared partner, interrupting one’s time with a shared partner, or attempting (successfully or unsuccessfully) to exert veto power over one’s own relationship.

...I think there are all kinds of ways to have soft vetos, or like softly limit another person’s relationship.... to make it so uncomfortable for your partner to be around that other person as to just like make it un-, make it really unpleasant for that other relationship to exist, and therefore sort of close it out of existence. (06)

#### Theme 4: Difficulties Related to Interactions Between Metamours (n = 47)

Participants talked about the costs that can stem from interactions between metamours, including the general time and emotional energy spent sharing space with and cultivating a good relationship with metamours or with a metamour’s other partners or wider community, as well as the work of navigating specific conflicts or difficult situations with metamours.

Yeah, it’s like a time-management thing at this point. And it’s also just like, oh this is like a whole other relationship, and with it comes just things that come with relationships, like conflict-resolution and dealing with differences in personality and communication styles and stuff like that. It’s like having, I mean you know, it’s like a regular friendship, but not... (16)

Participants also described ways in which it can feel unpleasant, engender extra work, or even limit the amount of time spent with a partner when one does not especially like or get along with a particular metamour, although a few talked about not liking a given metamour in ways that suggested that it was not a big problem for them.

There is a case where I have a relationship with someone and their partner is very abrasive.... there are some moments that are difficult, grating.... (Interviewer: Do you feel like that’s impacted at all your relationship with that partner that you share? Or is that not?) I think it’s impacted it in the way, the amount of time we could be spending together, yeah. (Interviewer: Is that your keeping some distance because of that?) I think I find myself, that I am. Yeah. (41)

In the opposite vein, participants sometimes described feelings of sadness or grief when a relationship ends and an enjoyable connection between former metamours changes, becomes complicated, or is lost entirely.

I guess some of the negative stuff that happens is when that relationship doesn’t work out for my, for a partner... and I was feeling all this compersion and such, then that can be hard, especially if it’s somewhat of a relationship I’m invested in... it’s like a secondary grief.... Part of it too is because like, when I do like a metamour... usually there is a dynamic between like, the three of us... And when their relationship fails.... It’s like, aw, this dynamic is lost. (30)

Participants also described some specific kinds of difficulties that could arise when metamours interact, including having to personally experience a metamour’s difficult feelings about oneself or one’s own relationship with a shared partner, witnessing relationship problems between a partner and metamour or even being drawn into them, dealing with metamours whose awareness of or approach to societal problems (e.g., racism) or identity issues (e.g., preferred pronouns) is very different from one’s own, or disagreeing with a metamour regarding how to practice CNM (e.g., how hierarchies should work, how well metamours should get to know each other, or how individuals should behave toward a partner when spending social time with partners and metamours).

...my metamour is feeling really jealous.... And because we’ve been getting close.... to learn now how jealous she is of me is actually, kind of hurts on a personal level, ’cause I think like, “I was your friend. I am your friend, aren’t I? How could you be jealous of me?” (47)

Difficulties between metamours were sometimes described as having the potential to lead to difficulties in the relationships with the shared partner, in particular when a shared partner takes on or is pulled into a mediating role between metamours, and in this way, this theme intersects with Theme 3, “difficulties in or undesired changes to one’s own relationship.”

...I had a conflict situation with a metamour where... I didn’t want to triangulate, I wanted to speak to them directly, and they did not want that.... So it resulted in me having an extended argument with my partner about it, because my metamour didn’t want to talk to me directly. Which was a very strange position to be in, to be having an extended argument with my partner, literally not about them. (29)

### Section 2: Perceived Benefits to Oneself or Things that Are Enjoyable for Oneself About Partners’ Other Partners/Relationships

#### Theme 5: Enjoyment of Compersion Feelings (n = 27)

Participants described experiencing enjoyable feelings of compersion. This theme in some ways intersects with most of the other themes that describe benefits to oneself (Themes 6, 7, and 8), each of which likely often involves enjoyable feelings. What is included in this theme are positive feelings that are not clearly associated with any of the specific benefits to oneself described in other themes, and whose main benefit is simply a positive feeling that the individual gets to enjoy.

Experiences of compersion were usually described in one of two ways. The first was a general feeling of being “happy for” a partner on account of their other relationship activities.

...most of the time when I feel compersion, it’s kind of like, this excitement that your partner’s doing something exciting. Like it feels like you’re genuinely happy for someone. (41)

...if somebody’s having a good time when I’m not there, then I’m happy for them, generally speaking.... If somebody whom I want to be, whom I care about, is happy, then it affects me. I get all happy too. (40)

The second was a more specific feeling of personal pleasure or vicarious enjoyment upon either witnessing a partner and metamour interacting or hearing about details of a partner and metamour’s relationship.

Sometimes when I’m feeling extra compersiony... sometimes I will ask [a metamour] about the relationship and what they’re—and I do get a lot of compersion, so I do kind of try to get in there and see what things they’re excited about... yeah I’m sure there’s some selfish aspect of just kind of getting more compersion, seeing what they’re excited about... (30)

Participants also occasionally referenced experiences of compersion in a general way, without describing their experience in enough detail to clearly fit into either of the above categories (e.g., one participant noted, “I wish that my metamour could feel compersion…. I feel compersion…” (47), but did not describe the experience of compersion).

#### Theme 6: Benefits and Positive Feelings Stemming from Partners Getting Their Needs Met by Others (n = 22)

Participants described being happy about a partner getting some of their needs met in other relationships, often describing these as an indirect benefit to themselves in that these were needs that they themselves could not or did not want to meet, either in general or in a particular instance.

...there have definitely been times when a metamour has saved my bacon.... times when I was in conflict with my [partner]... [metamour] would step in and be supportive of them in that conflict, which took some pressure off me, because it’s like, we can have more space, because you have somebody else to meet those needs. And so I’m indirectly benefiting. (34)

Some participants specifically described how partners getting some of their needs met by others benefited themselves by giving them more time to do other things or be alone.

...I have experienced metamours often times as relief from the emotional duties of being a partner… I am like, ridiculously capable of being alone and happy. And so that kind of plays into it, right? ’Cause like, it gets, it buys me alone time. (19)

Other participants described benefiting from and/or deriving pleasure from collaborating with metamours to meet the needs of a shared partner, which intersects with Theme 8, “benefits to oneself as a result of interacting with metamours.”

Yeah I prefer having some sort of relationship with a metamour, so that if something, like if a partner is sick, or I see that they’re having a difficult emotional time, something like that, I can reach out to them and be like, hey, I don’t have all of what partner needs to heal right now; is there anything that you can provide? And kind of be there together in that. And yeah, so being able to have that network, so that I’m not the only support person, and also so that I’m aware that I’m not the only support person, is really important. (17)

Responses describing perceptions of benefits to oneself as a result of a partner getting needs met by others also occasionally intersected with Theme 7, “benefits to one’s own relationship stemming from a partner’s other relationships” and Theme 9, “opportunities for personal growth as a result of sharing partners with others.”

Like selfishly speaking, nonmonogamy made the experience of [medical issue] profoundly better, not just because I had another partner, or other partners during that journey, but because [partner] had other partners, and she got support that I could not offer her. And it made her better, made her more durable, and we exited that experience with her and us, our connection, far stronger than it might have been. (32)

#### Theme 7: Benefits to One’s Own Relationship Stemming from a Partner’s Other Relationships (n = 26)

Participants described various ways in which a partner’s activities with a metamour could benefit their own relationship with that partner. Some participants described ways in which a partner’s other relationships could improve that partner’s happiness, well-being, or relationship skills in a way that benefited their own relationship.

...my metamour is like a net plus to my life, because they’re making my partner a richer person.... every time your partner goes through personal growth, you get some payoff from that, right? So a good metamour is a form of personal growth for your partner. (29)

Some participants described how a partner’s other dating activities could benefit themselves by bringing up opportunities to examine what they want in their own relationship, through observing what is happening in a partner’s other relationship.

...I can learn from watching my partner be different with one of their partners, right, to see that they’re capable of doing something differently than they do with me. And so there’s like a lot of growth opportunity, just because you want a different connection. (19)

And finally, some participants described specific instances of enjoyable or intimacy-enhancing interactions with partners as a result of the partner’s other relationships, including talking with a partner about their experiences with a metamour, intimate interactions inspired by a partner’s recent activities with a metamour, or supporting a partner through a difficult situation in another relationship.

If they [partner] come home from a date, and they’re all like, “oh my god that was so much fun,” usually there’s really good sex gonna happen right then, you know? (40)

There’s a part of me that, like I think one of my love languages is taking care of my people, so I feel so connected when I can help my person when they’re freaking out.... it makes me feel so loved when a person’s like, I’m struggling; can you help me with this? So even that hard part about the metamour stuff weirdly pulls me even closer to my people. (35)

#### Theme 8: Benefits to Oneself as a Result of Interacting with Metamours (n = 45)

Many participants described how metamours can become friends and mutually supportive community or even sexual/romantic partners. Some participants specifically described how a partner’s taste in partners tended to result in enjoyable metamour relationships for themselves, and a few mentioned that they had yet to meet a metamour that they didn’t like.

...you meet great people [by meeting metamours], like if you meet someone who’s great, then the people that they think are great are probably great. So it’s a very good predictor of interesting, lovely people. And I’ve actually several times made friends with a metamour that, that friendship lasted beyond the relationship. (20)

Some participants described deriving enjoyment from talking with a metamour about a shared partner or even simply getting to interact with another person who has a relationship with their partner.

...I’ve been able to talk with so many partners of partners. There’s kind of a joke, though, about that, which is, my little bit of a kink is, I’m a voyeur. So I cannot not want to meet and know and see and talk with my partner’s other partners. (44)

Participants also described several ways in which metamours can interact that directly benefit each others’ relationships: by cultivating and expressing positive sentiments about each others’ relationship with their shared partner, by providing direct information to each other about a shared partner via conversation and interaction, by providing indirect information for each other about a shared partner via knowing each other and observing each others’ interactions with that partner, or by providing helpful support during difficulties in each others’ relationship (although this latter was described as helpful at times, some participants also mentioned that such help should be sought or provided with care because it could end up having undesired consequences, and in this way, this theme intersects with Theme 4, specifically the element of being drawn into relationship difficulties between a partner and metamour). These benefits also intersect conceptually with Theme 7, “benefits to one’s own relationship stemming from a partner’s other relationships.”

Yeah no it’s, I love it [knowing metamours]. It’s good. I like, you can see what someone, what makes someone tick by who else is in their life, kind of. And I really like that. (20)

I have experienced this to some extent, but I also know a lot of people have, and it can be dangerous territory, but [metamours] can offer insight into your relationship issues sometimes. I do think that’s a slippery slope in terms of.... they’re not going to be objective, but that being said, sometimes they do have really valuable insight, so I think it’s a very case-by-case basis. (43)

Finally, some participants described specific ways in which metamours can be resources for each other outside of their connection with their shared partner, through social support, sharing skills or knowledge, or sharing childcare and other householding activities.

... I just can also trust all of my metamours, like if I need something, not even for one of my partners, I know I could call the metamour and just be like, hey could we talk? As a friend. So because my partners are dating cool and interesting and amazing people, my support network has extended. (39)

#### Theme 9: Opportunities for Personal Growth as a Result of Sharing a Partner with Others (n = 20)

Participants talked about how sharing a partner with others brings up opportunities for personal growth, and this was described in two distinct ways.

First, some participants described how sharing a partner can result in emotional growth, by providing opportunities to become a more well-rounded, mindful, or compassionate person, or to cultivate self-love or security that is based within oneself.

Yeah, I mean, I think that one of the best things about nonmonogamy as a practice is that it requires you think and work through jealousy in ways that almost nothing in our society asks you to do.... And I know that the work I have done to sort of de-fang jealousy inside myself is very connected to the work I have done to develop my own self-love. And I think in some ways my self-love is stronger than had I never had to do that. (31)

And second, some participants talked about how sharing a partner brings up opportunities to improve one’s self-knowledge and relationship skills, by providing opportunities to become more aware of one’s own feelings and needs, where they come from, and how to articulate them effectively to partners.

I have definitely, like polyamory, I think, teaches you a lot about yourself, whether you want it to or not.... [partner] taught me a lot about myself, and in that, I had this bad feeling about this [metamour], and I went away with it for like [amount of time] and just didn’t see [partner] for [amount of time] while I thought about it, and then came back to him and was like, I felt this, it was because of this, this is what I need. (24)

## Discussion

Our results demonstrate that individuals who practice CNM experience a complex set of both costs and benefits to themselves stemming from their partners’ other relationships, findings that confirm and extend existing research. Supporting the argument of Watson and Lubrano (2021), our findings indicate that, far from being a cost that is tolerated in exchange for having multiple partners oneself, partners’ other relationships can impact oneself in a number of positive and negative ways. On the positive side, we found evidence of CNM relationships supporting and enhancing each other (e.g., Bartell, 1970; de Visser & McDonald, 2007; Jenks, 1998; O’Byrne & Haines, 2021; Ramey, 1975; Rubel & Bogaert, 2015; Thouin-Savard, 2021; Watson, 1981; Wood et al., 2021a), of enjoyable compersion as a result of partners’ other relationships (e.g., Deri, 2015; Ramey, 1975; Thouin-Savard, 2021; Wolfe, 2003), and of direct benefits that metamours can provide to each other (e.g., Bove, 2017; Ritchie & Barker, 2006; Sheff, 2014; Thouin-Savard, 2021; Watson & Lubrano, 2021). On the negative side, we found evidence of partners’ other relationships limiting or damaging one’s own (e.g., Cohen, 2015; Rubel & Bogaert, 2015; Rubinsky, 2018; Weitzman, 2006; Wolfe, 2003), of difficult feelings such as jealousy (e.g., de Visser & McDonald, 2007; McLean, 2004; Ramey, 1975; Rubinsky, 2018; Watson, 1981), and of ways in which relating to metamours can be difficult or painful (e.g., Deri, 2015; Rubinsky, 2018; Thouin-Savard, 2021). Our findings also go beyond the current literature in specific ways that we discuss in detail below.

First, we note one striking and unexpected aspect of our results: the themes that describe costs and the themes that describe benefits can be broken down into pairs that appear to be, in a general sense, mirror images. Thus, feelings about sharing partners could be painful (e.g., jealousy; Theme 1) or pleasurable (i.e., compersion; Theme 5); one could “miss out” on time/activities with a partner (Theme 2) or could benefit from partners getting needs met in other relationships (Theme 6); one’s own relationship could be damaged (Theme 3) or enhanced (Theme 7) by a partner’s other relationships; and interactions with metamours could be difficult and time-consuming (Theme 4) or beneficial and enjoyable (Theme 8).

These pairings suggest that our themes may reflect the positive and negative sides of some fundamental realities involved in sharing partners with others, which are also some of the fundamental realities of CNM as a practice: dividing one’s time, activities, and commitments among multiple partners, who then are connected to each other via interrelated relationships, means that relationships can affect each other both positively and negatively, individuals can get along well or poorly, and both positive and negative feelings can result. Our findings point to the importance of future research that can illuminate the conditions under which individuals in CNM relationships experience and navigate these relationships positively versus negatively.

The final theme we present (Theme 9, “opportunities for personal growth as a result of sharing a partner with others”) has no parallel “cost” theme, perhaps because those who found or would find CNM to be detrimental to their personal growth do not engage in it and are not represented in this sample. However, this theme may reveal an important part of what ultimately makes the benefits of CNM outweigh the costs for those who practice it. Responses in this theme often described personal growth stemming from or intertwined with things that are perceived as difficult about sharing partners. These responses suggest that it is not simply that the pros outweigh the cons for those who practice CNM, but that the cons also contain a “silver lining” that is experienced as a benefit in the long run (see Deri, 2015; Weitzman, 2006).

### Feelings Experienced in CNM Relationships

Our findings with regard to “struggling with unpleasant feelings” (Theme 1) support the notion (McLean, 2004; Rubinsky, 2018; Wolfe, 2003) that individuals experience a broad range of such feelings, which are not all reducible to “jealousy.” Indeed, some of our participants drew explicit distinctions between jealousy and other negative feelings (e.g., insecurity, inadequacy, envy, or fear of the unknown). The range and nuance in such feelings deserves more attention in future work. Our findings also suggest that these feelings often involve an element of comparison between oneself and a metamour (Deri, 2015; Thouin-Savard, 2021); future work might explore comparison and the avoidance of comparison as an element influencing the feelings experienced in CNM relationships. Additionally, in line with Thouin-Savard’s (2021) findings, some of our participants described ways in which jealousy could arise in part from habits and expectations learned from monogamous culture. Thouin-Savard (2021), Bove (2017), and Watson and Lubrano (2021) suggest that such expectations and their accompanying feelings can be unlearned, and if so, such unlearning might be investigated as an element influencing feelings experienced in CNM relationships.

Our findings regarding “enjoyment of compersion feelings” (Theme 5) support the notion that such feelings are both common and important in CNM (Deri, 2015; Flicker et al., 2021; Mogliski et al., 2019; Ritchie & Barker, 2006; Rubinsky, 2018; Thouin-Savard, 2021; Wolfe, 2003) and deserve more focus; cultivating such feelings may be one of the major elements contributing to happy CNM relationships (Ferrer, 2019; Thouin-Savard, 2021). Our findings also suggest that compersion can be experienced in different ways; our participants described two distinct kinds of experiences: a broad feeling of being “happy for” a partner’s happiness in another relationship and a more personally focused feeling of vicarious pleasure when witnessing or hearing about a partner’s other relationship(s). This distinction is reflected in Thouin-Savard’s (2021) comprehensive exploration, which describes “attitudinal” compersion and “embodied” compersion; Duma (2009) similarly describes “trait” versus “state” compersion.

### Effects of Relationships on Each Other

Time is a finite resource; dividing one’s time and committments among multiple partners is a major element of CNM. However, our findings and others suggest that both positive and negative outcomes can result from such time divisions. “Having less with a partner because of the existence of the partner’s other relationships,” describes costs including “missing out” on time or particular activities with a partner (Ramey, 1975; Rubinsky, 2018; Wolfe, 2003) or wanting a kind of relationship that is not available due to a partner’s other relationships. However, as demonstrated in Theme 6, “benefits and positive feelings stemming from partners getting their needs met by others,” the time that a partner spends with their other partner(s) is sometimes instead perceived as a benefit to oneself, by creating “alone time,” releasing oneself from meeting a partner’s needs (Deri, 2015; Thouin-Savard, 2021), or allowing collaboration with metamours to meet a partner’s needs (Bove, 2017; Watson & Lubrano, 2021). Indeed, one of the perceived benefits to oneself of having multiple partners is having one’s own various needs met in different relationships (Arter & Bunge, 2023; Moors et al., 2017; Wood et al., 2021a). Our finding that some individuals perceive benefits to themselves from their *partners* getting needs met in other relationships suggests that for at least some who practice CNM, each individual getting needs met across multiple relationships may be experienced as a net benefit for all parties. Future work might explore this possibility, along with the circumstances that might promote such experiences.

In a similar vein, Theme 3 (“difficulties in or undesired changes to one’s own relationship”) and Theme 7 (“benefits to one’s own relationship stemming from a partner’s other relationships”) describe negative and positive ways in which events or decisions within a partner’s other relationship(s) could “spill over” and influence one’s own relationship for better or worse. These effects can be complicated; difficulties in a partner’s other relationship could lead to difficulties in one’s own, but could sometimes lead instead to feeling closer to that partner via providing support (and see Thouin-Savard, 2021, for similar documentation of such complex effects). One’s experience of such situations may depend on the specifics of the situation, and further investigation of such influences might result in a better understanding of the complex ways in which CNM relationships can impact each other, and perhaps ultimately some indication of how to maximize positive influences and minimize negative ones.

Our results in Theme 7 (“benefits to one’s own relationship”) are also in line with previous reports documenting the possibility that personal growth via one relationship could result in growth within a different relationship (Thouin-Savard, 2021; Wood et al., 2021a). One of the reasons often reported for engaging in CNM is the seeking of self-growth and self-expanding experiences (Moors et al., 2017; Wood et al., 2021a); our results add to this picture the notion that one’s own relationship might benefit from a partner’s seeking of growth experiences in other relationships, and suggest one possible mechanism: Witnessing a partner in another relationship can provide opportunities to examine how one would like one’s own relationship with that partner to be.

On the negative side, Theme 3 (“difficulties in or undesired changes to one’s own relationship”) documents damage to or even the loss of a relationship as the result of another relationship (Cohen, 2015; Rubel & Bogaert, 2015; Wolfe, 2003), and we found that such loss could originate in any of the involved individuals: a partner, a metamour (Rubinsky, 2018; Thouin-Savard, 2021; Weiztman, 2006), or even oneself. Again, the circumstances leading to such outcomes deserve further focus.

### Interactions Between Metamours

Theme 4 (“difficulties related to interactions between metamours”) and Theme 8 (“benefits to oneself as a result of interacting with metamours”) contribute to an emerging understanding of relational dynamics between metamours. Both difficulties and benefits were mentioned by a large majority of our participants and each included a broad array of elements, suggesting that metamour relationships, unique to CNM, can be nuanced and complex and may at times strongly influence the happiness of the romantic relationships that create them. Some of the elements in these two themes mirror previous research and others may not have been previously documented. On the positive side, our participants described metamours providing each other with practical assistance and resources (Sheff, 2014; Watson & Lubrano, 2021); developing important relationships with each other (Bove, 2017; Ritchie & Barker, 2006; Thouin-Savard, 2021; Watson & Lubrano, 2021) and even becoming lovers (Thouin-Savard, 2021); directly supporting each others’ relationships via expressing support, providing information about shared partners, or assisting each other in relationship difficulties; or simply enjoying discussion of their shared partner. On the negative side, our participants described metamours comparing themselves with or feeling competitive toward each other (Deri, 2015), disliking or distrusting a particular metamour (Rubinky, 2018; Thouin-Savard, 2021), spending time and energy to cultivate metamour relationships and deal with difficult situations (Watson & Lubrano, 2021), having to experience a metamour’s difficult feelings about their own relationship, witnessing or being drawn into conflicts between partners and metamours, disagreeing with a metamour regarding how to practice CNM, or experiencing loss of a metamour relationship when a romantic relationship ends. In future work, we hope to more extensively describe relational dynamics between metamours, and how these dynamics influence and interact with those within romantic relationships.

Overall, our findings suggest that a complex set of mechanisms likely influences whether a given situation will result in positive or negative outcomes for a given CNM relationship, and indicate several avenues for exploration of the kinds of practices and circumstances that tend to result in happy and successful CNM relationships. We note that family systems literature would likely prove useful in future approaches to this topic (Bove, 2017).

### Limitations of the Current Work

This study has some important limitations. First, the fact that our sample was specifically recruited to include participants with experience interacting with metamours means that this sample is strongly skewed toward those who meet and spend time with metamours. There may be many people who practice CNM in such a way that metamours rarely interact (sometimes referred to as “parallel polyamory,” e.g., Mahler, 2016; Sanchez, 2019), and the costs and benefits of CNM for these individuals would likely look different, in particular the costs and benefits of interacting with metamours, but we additionally speculate that some kinds of negative and positive feelings might be experienced less often when metamours are rarely witness to each others’ relationships, and that the perceived pros and cons of partners’ other relationships might center more on sharing partners’ time and partners getting needs met by others.

Additionally, we asked participants about their experiences with metamours, but we did not specifically and systematically ask participants to delineate the costs and the benefits to themselves of having metamours with whom they share their partner(s). This was because we did not set out with the goal of examining the costs and benefits of partners’ other relationships, but rather developed this major set of themes upon exploring the data. Thus, there may be additional insights that we did not capture, and it may be that some of these themes would become more nuanced and might be mentioned more often by participants who were more directly asked.

A final limitation, and a direction for further research, is that the current work does not take up the question of what kinds of circumstances contribute to an individual experiencing a partner’s other relationships as costing or benefiting themselves. The suggestion that these costs and benefits are mirror images of each other and that they reflect some of the basic realities of CNM points to the idea that there may be circumstances that lead an individual to experience a partner’s other relationships as generally beneficial to themselves, or as a cost to themselves. Describing the circumstances that influence these perceptions would contribute not only to the understanding of how CNM as a practice functions, but also to the beginnings of a body of research examining how to practice CNM in ways that result in strong and happy relationships. There is an extensive body of research describing monogamous relationships and how to help individuals engage in them in effective and rewarding ways (e.g., Gabb & Fink, 2015; Gurman, 2008), but as yet little research that has aimed at similarly benefiting those who practice CNM (but see Schechinger et al., 2018; Stavinoha, 2017; and Weitzman, 2006).

### Conclusions

Evidence indicates that CNM is in no way inherently flawed as a way of engaging in intimate relationships, but neither is it inherently superior to monogamy (e.g., Cohen, 2015; Ferrer, 2018b; Moors et al., 2017; Ramey, 1975; Rubel & Bogaert, 2015; Sheff, 2014; Thouin-Savard, 2021; Wood et al., 2021a). Our findings document some of the complexities of CNM relationships and show that they can be both uniquely challenging and richly rewarding, in interrelated ways. Given that a sizable minority of individuals engage in CNM (e.g., Fairbrother et al., 2019; Levine et al., 2018) and that some appear to prefer it strongly over monogamy (Arter & Bunge, 2023), further research examining the nuances of these relationships, and how to engage in them in ways that are likely to result in satisfying and happy relationships, is indicated. We hope that our findings contribute material and inspiration for research that has begun to move beyond documentation of the existence and viability of CNM as a practice, and into exploration of the complex interpersonal dynamics at play in CNM relationships.

## Data Availability

De-identified data (direct quotes from participants) underlying the results of this study are available from the first author on request, subject to minor redaction due to privacy concerns of specific participants.

## Appendix 1: Interview Questions Eliciting Relevant Responses from Participants

Participants often made statements relevant to the current paper in response to three of the opening questions: “What’s good and what’s hard about knowing and having connections with metamours?” “Do you feel that you personally benefit from having and/or knowing metamours, and if so, how?” and “Getting to know metamours, has that been a more positive or more negative experience, or has it varied?”

Relevant statements were also often obtained from questions on the topic of feelings about metamours, discussed by 21 participants and including questions about how participants define and relate to the concepts of jealousy and compersion, how often they feel each of these types of feelings and in what kinds of situations, how they deal with such feelings, how often and how they communicate about such feelings with partners and/or metamours, what kinds of things partners can do to help each other with difficult feelings, and whether there are things that they and partners proactively do to ease difficult feelings and/or promote positive ones. (Many participants who were not asked these questions nevertheless talked about jealousy and compersion in the course of answering questions in other topic areas.)

Relevant statements also sometimes came from questions about privacy and information-sharing, asked of 13 participants and including questions about how much participants know about the details of partners’ other relationships and about their metamours’ lives, what and how much they prefer to know, how much they share with partners about their own other relationships, what kinds of information they prefer to keep private, and how they navigate conflicts with partners regarding what or how much to share.

Although there was a set of questions about difficult situations, only five participants were asked questions from this topic list. However, many participants described difficult situations in the course of answering questions from other topic areas. Questions about difficult situations included questions about whether participants had ever disliked a metamour or had specific conflicts with a metamour, and if so how they handled those situations; whether participants had been in situations with metamours that were influenced by privilege differences (e.g., race and class) and how they dealt with those; whether participants had had metamours who were new to CNM and how that worked out; and what was the most difficult situation with a metamour that participants had encountered, how they thought the situation had arisen, what they did to address the situation, and how the situation unfolded over time.

And finally, relevant statements also came from particular participants’ responses to a variety of other questions throughout the interview, including questions about how it is for participants when they gain a new metamour, how well they prefer to know metamours, how it is for participants to lose a metamour, what they think about hierarchies and veto power in CNM relationships, whether participants have ever started dating a metamour, and what kinds of etiquette participants prefer when they are in the same place with partners and metamours.
